# Extensive transcriptome changes during seasonal leaf senescence in field-grown black cottonwood (*Populus trichocarpa* Nisqually-1)

**DOI:** 10.1038/s41598-020-63372-2

**Published:** 2020-04-20

**Authors:** Haiwei Lu, Michael I. Gordon, Vindhya Amarasinghe, Steven H. Strauss

**Affiliations:** Department of Forest Ecosystems and Society, Oregon State University, Corvallis, Oregon USA

**Keywords:** Plant development, Plant stress responses

## Abstract

To better understand the molecular control of leaf senescence, we examined transcriptome changes during seasonal leaf senescence in *Populus trichocarpa* Nisqually-1, the *Populus* reference genome, growing in its natural habitat. Using monthly (from May to October) transcriptomes for three years (2009, 2015, and 2016), we identified 17,974 differentially expressed genes (DEGs; false discovery rate <0.05; log-fold change cutoff = 0) from 36,007 expressed *Populus* gene models. A total of 14,415 DEGs were directly related to transitions between four major developmental phases – growth, senescence initiation, reorganization, and senescence termination. These DEGs were significantly (*p* < 0.05) enriched in 279 gene ontology (GO) terms, including those related to photosynthesis, metabolic process, catalytic activity, protein phosphorylation, kinase activity, pollination, and transport. Also, there were 881 differentially expressed transcription factor (TF) genes from 54 TF families, notably bHLH, MYB, ERF, MYB-related, NAC, and WRKY. We also examined 28 DEGs known as alternative splicing (AS) factors that regulate AS process, and found evidence for a reduced level of AS activity during leaf senescence. Furthermore, we were able to identify a number of promoter sequence motifs associated with leaf senescence. This work provides a comprehensive resource for identification of genes involved in seasonal leaf senescence in trees, and informs efforts to explore the conservation and divergence of molecular mechanisms underlying leaf senescence between annual and perennial species.

## Introduction

Leaf senescence is the programmed death of foliage that enables nutrient mobilization from leaf into seeds in annual plants, or into buds, trunk and roots in perennial plants. It is an essential mechanism of plant adaptation and survival^1,2^. The onset of leaf senescence can be triggered by endogenous factors, such as plant age, reproduction, and the level of phytohormones, and exogenous stimuli, such as prolonged-darkness, extreme temperature, and other abiotic stresses^3–5^. During leaf senescence highly coordinated, sequential changes occur in gene expression, cell structure, and metabolism, which have been subdivided into three stages: initiation, reorganization, and termination^3^. The initiation phase is hallmarked by large shifts in gene expression. Such alteration leads to chloroplast breakdown and the replacement of carbon assimilation, including photosynthesis, by catabolism of chlorophyll and other macromolecules that culminate during the reorganization phase. The profound metabolic changes ensure nutrient remobilization before the disruption of mitochondria and nucleus in the terminal phase.

In the past decade, transcriptome analysis has enabled the identification of a large repertoire of senescence-associated genes (SAGs) and facilitated understanding of the molecular control of leaf senescence, particularly response to endogenous stimuli, aging and seed-filling. For example, in the model plant species *Arabidopsis thaliana* (*Arabidopsis*), Breeze *et al*.^6^ produced a set of time-course microarray data that examined changes in protein-coding messenger RNA (mRNA) expression during leaf development and senescence. Woo *et al*.^7^ generated a set of multidimensional leaf transcriptome data using RNA sequencing (RNA-Seq). They examined changes in not only mRNAs but also non-coding RNAs (ncRNAs) transcribed from nuclear, chloroplast, and mitochondrial genomes. In addition to *Arabidopsis*, transcriptome data has been produced from a number of annual species, including cotton (*Gossypium hirsutum*), soybean (*Glycine max*), wheat (*Triticum aestivum*), maize (*Zea mays*), and rice (*Oryza sativa*)^8^. In agreement with the apparent physiological changes, these temporal transcriptome profiling studies found downregulation of genes involving in anabolic processes, such as chlorophyll biosynthesis and photosynthesis, and upregulation of genes participating in catabolic processes, such as cell wall degradation and lipid catabolism. Transcription factors (TFs), such as NAC, WRKY, bHLH, and bZIP, were found to have key roles in regulating leaf senescence^6,9–11^. Environmental signal-induced senescence has much in common with age/reproduction-dependent senescence, yet it requires the coordination of different sets of genes, especially during the onset and initiation stages. For example, Buchanan-Wollaston *et al*.^12^, and Guo and Gan^13^ revealed 1,233 differentially expressed and 474 upregulated genes, respectively, that are uniquely involved in dark-induced senescence in *Arabidopsis*.

Despite the large volume of data generated from annual plants, perennials, which exhibit seasonal leaf senescence upon shortened day length^14^, remain largely unvisited. An initial effort to investigate transcriptome changes during seasonal leaf senescence was made by Andersson *et al*.^15^ using a 13,500 transcript microarray derived from a single aspen (*Populus tremula*) tree in a natural stand. Another study was performed by Wen *et al*.^16^ using microarray and RNA sequencing (RNA-Seq). The authors profiled transcriptome changes during leaf development in Formosan gum (*Liquidambar formosana*). Both studies suggested that seasonal leaf senescence in trees share common regulatory mechanisms with leaf senescence in annual plants, in terms of the major changes in gene expression and the key TF regulators that were identified.

**Figure 1:**
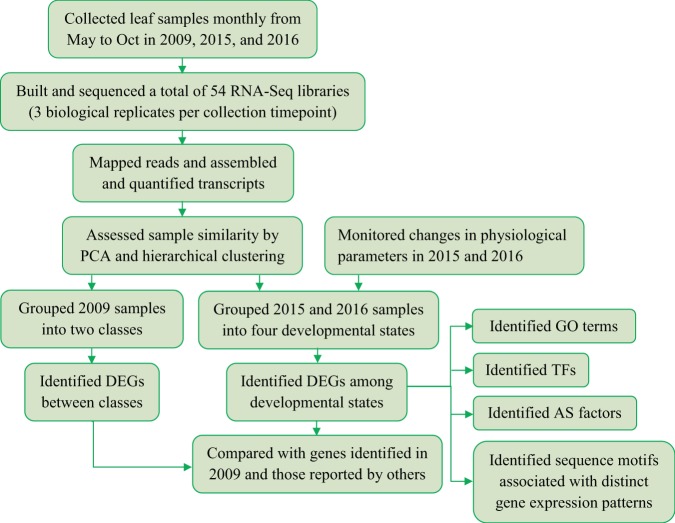
Overview of experimental strategy. Abbreviations used: PCA = principal component analysis, DEG = differentially expressed gene, GO = gene ontology, TF = transcription factor, AS = alternative splicing.

To extend the knowledge of gene expression changes during leaf senescence in perennial plants in a natural environment, we examined transcriptome changes during leaf growth and senescence in three field-grown *P. trichocarpa* trees for three years using RNA-Seq (Fig. 1). Specifically, the hypotheses that we aimed to test include: (1) due to the in-depth sampling and sequencing, our dataset will enable the identification of a large number of SAGs that were not previously reported in perennials. (2) Because seasonal senescence in *Populus* and age-dependent senescence in *Arabidopsis* are triggered by different factors, a unique set of differentially expressed genes (DEGs) is associated with the onset of senescence in each case. (3) Previous studies have revealed common regulatory networks controlling the progression of senescence across plant species, therefore the DEGs, gene ontologies (GOs), and TFs identified in this study will strongly overlap with those associated with leaf senescence in other plant species. (4) Because alternative splicing (AS) has been known to regulate plant defense and environmental fitness, a number of AS factor encoding genes will be recognized from the DEGs identified in this study. (5) In field grown trees, three simultaneously occurring processes, seasonal leaf senescence, bud dormancy, and cold acclimation, are induced by common environmental stimuli, shortened day length and low temperature, therefore there will be overlaps between DEGs identified in this study and dormancy-associated genes (DAGs) and cold-acclimation-associated genes (CAGs) previously reported in *Populus*. Directed by these hypotheses, we report extensive transcriptome remodeling that both support and extend expectations from previous publications.

## Methods

### Plant material

**Figure 2:**
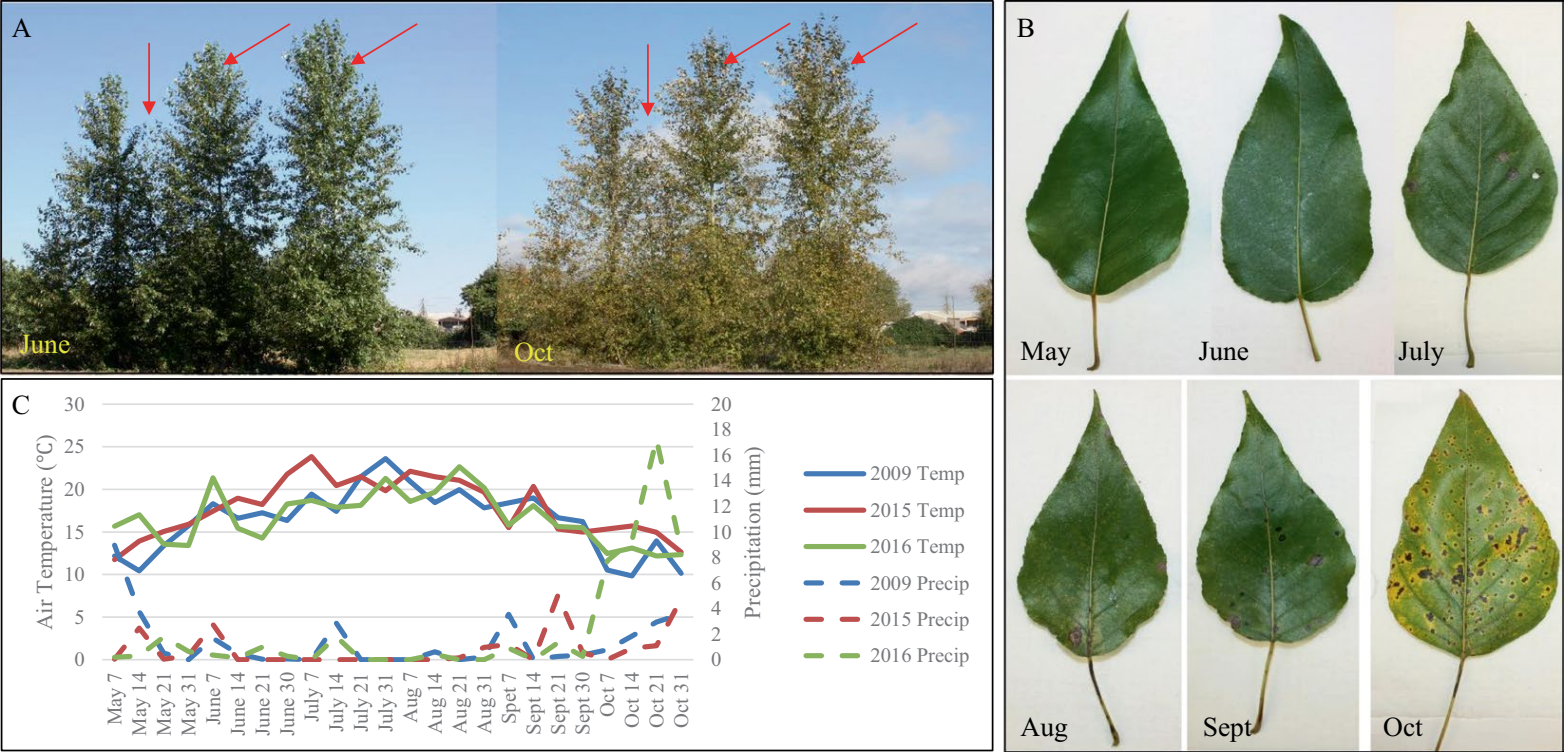
Plant materials and collection condition. (**A**) Three *Populus trichocarpa* trees (clone Nisqually-1, planted in Corvallis OR), indicated by red arrows, used for 2015 and 2016 collections. Images taken on June 30, 2015 and October 29, 2015, respectively. (**B**) Leaf samples collected in 2015. Images taken on May 29, June 30, July 29, August 31, September 30, and October 29, in 2015, respectively. (**C**) Weekly average temperature and precipitation in Corvallis OR during the sample collection period.

*P. trichocarpa* (clone Nisqually-1) trees planted on a field site in Corvallis, OR, USA were used in this study. The three trees used for 2009 collections were planted in 2003, while the three trees used for 2015 and 2016 collections (Fig. 2A) were planted in 2009. In all three collection years, we collected leaf samples in afternoons (ranging from 2 pm to 4 pm, depending on collection date) at the end of each month from May to October, specifically, on May 29, June 28, July 28, August 27, September 27, and October 28 in 2009, May 29, June 30, July 29, August 31, September 30, and October 29 in 2015, and May 31, June 30, July 27, August 30, September 29, and October 28 in 2016. For each tree, two fully expanded leaves (Fig. 2B) from branches on the north side of the tree 5 to 6 feet off the ground were cut using sterile scalpel blades, immediately placed in liquid nitrogen, and stored at −80 °C until they were used for RNA isolation. Average temperatures and precipitations over the collection period (obtained from Hyslop Weather Station, https://agsci.oregonstate.edu/hyslop-weather-station) are shown in Fig. 2C.

### Physiological data collection and analysis

At each collection timepoint in 2015 and 2016, we monitored changes in two senescence-associated physiological parameters: leaf chlorophyll content index (CCI), and electrolyte leakage index (ELI). The CCI was measured using a hand-held SPAD meter (Konica Minolta SPAD-502 Plus; Minolta, Osaka, Japan). Eight fully expanded leaves (from branches on the north side of the tree 5 to 6 feet off the ground, same as the ones collected for RNA isolation) were measured per tree, with three SPAD measurements per leaf. Three of these leaves were collected and placed on ice for 30 minutes before they were processed in lab for electrolyte leakage measurement adapted from Woo *et al*.^17^. In brief, a total of 15 leaf discs per leaf were cut using a clean hole punch, then immersed in 10 ml of distilled deionized water (ddH_2_O) in a 15 ml microcentrifuge tube at room temperature with gentle shaking for 24 hours, after which the initial conductivity was measured. Total conductivity was determined after boiling leaf discs for 10 minutes and allowing cool down to room temperature. Conductance, the indicator of ELI, was calculated by dividing the initial conductivity by the total conductivity. We performed ANOVA (R version 3.5.2) to examine differences in the physiological parameters among collection timepoints from each year.

### RNA-Seq library preparation and sequencing

Total RNA was isolated from leaf samples using a CTAB method^18^, followed with DNase I treatment to remove contaminating genomic DNA (Qiagen, Valencia, CA, USA). The quantity and integrity of RNA samples were examined using a NanoDrop 1000 (Thermo Fisher Scientific, Waltham, MA) and a Bioanalyzer 2000 (Agilent Technologies, Santa Clara CA) before RNA-Seq library construction and sequencing at Center for Genome Research and Biocomputing at Oregon State University (Corvallis, OR, USA). For samples from 2009, we performed mRNA enrichment using Oligotex mRNA Mini Kit (Qiagen, Valencia, CA, USA) and constructed libraries following the method reported by Filichkin *et al*.^19^. The resulting libraries were sequenced on an Illumina GAIIx instrument with paired-end 50 bp runs. Similarly, for each sample collected in 2015 and 2016, we performed mRNA enrichment and built strand-specific RNA library preparation using PrepX polyA mRNA Isolation Kit and PrepX RNA-seq for Illumina Library Kit (both from WaferGen Bio-Systems Inc, Fremont, CA, USA), respectively. The libraries were then sequenced on an Illumina HiSeq. 3000 instrument with 9 to 10 samples per lane and single-end 150 bp runs. The sequencing files have been deposited in the National Center for Biotechnology Information (NCBI) database (BioProject: PRJNA597006).

### Assessment of RNA-Seq data and identification of DEGs

We performed transcript assembly and mapping to the *P. trichocarpa* genome *v3.1*^20^ (downloaded from Phytozome *v12.1*) using the HISAT2-StringTie pipeline^21,22^. Using the Bioconductor R package ‘DESeq. 2’^23^, we first normalized the HISAT2-StringTie pipeline generated read counts. Next, we explored similarities among monthly collections from three years using principal component analysis (PCA) and hierarchical clustering, which directed us to examine 2009 data separately from 2015 and 2016 data, and grouped monthly collections into different states (see Results). We then conducted negative binomial Wald tests for differential gene expression analysis with false discovery rate (FDR) less than 0.05 and log-fold change (LFC) cutoff of 0.

### Validation of transcript abundance with quantitative RT-PCR

To validate gene expression reflected by DESeq. 2-normalized RNA-Seq counts, we selected four genes and examined their transcript levels in July, August, September, and October in 2015, using quantitative RT-PCR (qRT-PCR). The four selected genes were Potri.011G079500, Potri.001G348900, Potri.015G099200, and Potri.001G374800, which were homologs of *Arabidopsis CHLOROPHYLL-PROTEIN COMPLEX II SUBUNIT B1* (*LHB1B1*, AT2G34430), *CARBONIC ANHYDRASE 1* (*CA1*, AT3G01500), *WRKY DNA-BINDING PROTEIN 75* (*WRKY75*, AT5G13080) and *GLUTAMATE RECEPTOR 2.7* (*GLR2*, AT2G29120). The *Populus ELONGATION FACTOR1-BETA* gene (*PtEF1-beta*, Potri.009G01860) was used as reference genes. All gene-specific primers were designed using PRIMER3PLUS^24^, apart from those for amplifying *EF1- beta*, which were reported by Wang *et al*.^25^. The efficiency of each primer pair was evaluated with standard curve before performing qRT-PCR (Supplementary Table S1). Complementary DNA (cDNA) samples were synthesized from the same RNA samples used for RNA-Seq with SuperScript III Reverse Transcriptase (Invitrogen, Carlsbad, CA, USA). PCR were performed on a StepOnePlus real-time PCR system (Applied Biosystems, Foster City, CA, USA). The amplification condition consisted of an initial denaturation at 95 °C for 10 minutes, 40 cycles of 95 °C for 15 seconds, 57 °C for 15 seconds, and 72 °C for 20 seconds, followed by melt-curve analysis with a temperature increase of 0.3 °C per second. Three biological replicates were used for each examined collection timepoint; two technical replicates were used for each reaction. The relative gene expression was determined using the ∆C_t_ method. Pearson correlation coefficients between DESeq. 2-normalized RNA-Seq counts and qRT-PCR-measured transcript levels were calculated based on tree means.

### Functional analysis of DEGs identified from 2015 and 2016 RNA-Seq data

Due to large within-month variance observed in 2009 collections (see Results), we primarily focused on DEGs identified from 2015 and 2016 RNA-Seq data for downstream analysis. To explore the composition of these DEGs, we first assigned them into different GO groups according to the agriGO *v2.0* database^26^. We then identified known TF genes from the DEGs according to the *P. trichocarpa* TF database in PlantTFDB v4.0^27^ (http://planttfdb.cbi.pku.edu.cn/). Similarly, we identified AS factor encoding genes from the DEGs based on the annotation of *P. trichocarpa* genome *v3.1*. Expression profiles of genes of interest were plotted using R package ‘pheatmap’.

### Identification of overrepresented sequence motifs

To identify sequence motifs associated senescence, we performed fuzzy C-means clustering analysis using the R package ‘e1071’^28^ and clustered DEGs from 2015 and 2016 data based on their expression patterns. In fuzzy C-means clustering, the input dataset was grouped into n clusters. Every datapoint was related to each of the n clusters with varying degrees of belonging (*i.e*., membership, on a scale ranging from 0.0 and 1.0). Datapoints that located far from the center of a cluster had a low degree of belonging to that cluster and therefore low membership values. Each datapoint was assigned into the cluster to which it has the highest membership^29^. For genes with membership scores higher 0.80, we used 2 kb of upstream sequences relative to their transcription start sites, and performed sequence motif enrichment using two motif finding tools, HOMER and MEME, available at KBase^30^ (https://kbase.us/). To identify a high-confidence subset of motifs, we compared the lists of sequence motifs generated by HOMER and MEME using the Ensemble algorithm at KBase, with a threshold of 0.8 and a proportion of 1.0. We also compared the high-confidence motifs against known motif database *Arabidopsis*DAP (v1) using Tomtom Motif Comparison Tool^31^ (available at MEME Suite v5.0.5) with default settings.

### Comparison of DEGs with Venn diagrams

We further explored the unions and intersections of DEGs identified in several different cases using Venn diagrams. First, we compared 2015/2016-based DEGs with 2009-based DEGs. Then, directed by our hypotheses (2) and (5) (see Introduction), we compared 2015/2016-based results with three published studies, which reported DEGs in response to age-dependent leaf senescence^7^, the transition from para-dormancy to endo-dormancy^32^ and low temperature treatment at 4 °C^33^, respectively. Because the study conducted by Woo *et al*.^7^ was performed in *Arabidopsis*, we compared against their database using *Arabidopsis* gene models represented by the DEGs identified in this study. In all cases, results were visualized using four-way Venn diagrams generated with InteractiVenn^34^, or weighted three-way Venn diagrams produced with the R package ‘eulerr’^35^.

## Results

### Physiological changes during collection period

**Figure 3:**
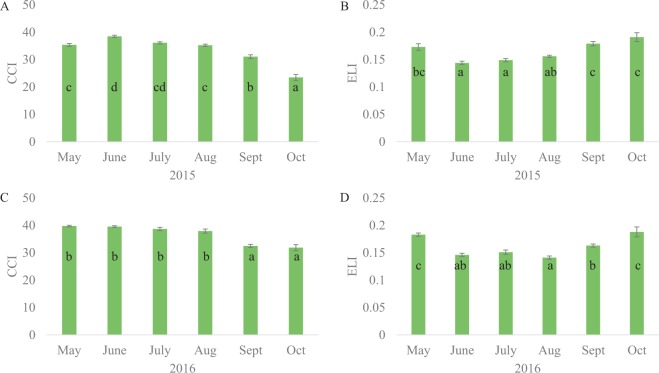
Changes in leaf chlorophyll content index (CCI) and electrolyte leakage index (ELI) from May to October in 2015 (**A**,**B**) and 2016 (**C**,**D**). Bars indicate averages, error bars indicated standard errors (SEs), and letters indicate ANOVA suggested significant levels.

We observed statistically significant (*p* < 0.05) changes among collections for CCI and ELI in 2015 and 2016. In both years, CCI remained high from May to August, then significantly diminished starting in September (Fig. 3; Supplementary Table S2). It continued decreasing from September to October in 2015, but kept at the same level in these two months in 2016. ELI was significantly reduced from May to June, and did not increase until September in both years. In 2016, ELI kept increasing from September to October. These changes indicated that our collections spanned highly differentiated physiological states during leaf development and death.

### Overview of RNA-Seq data from three years

We built and sequenced a total of 54 RNA-Seq libraries with three biological replicates per collection timepoint. For each library, we obtained 12.4 million to 65.1 million raw reads from Illumina sequencing, and 6.0 million to 41.1 million mapped reads from HISAT2-based mapping (Supplementary Table S3). StringTie-based transcript assembly and quantification revealed that a total of 36,007 gene models were expressed during the collection period (Supplementary Table S4). Principal component analysis (PCA) plot of DESeq. 2-normalized read counts showed that 2009 collections had larger variations compared with 2015 and 2016 collections (Supplementary Fig. S1), we therefore analyzed 2009 data separately from the other two years.

### Grouping of the 2009 collections into two classes

Both PCA plot and heatmap of sample-to-sample distances revealed large within-month variance in 2009 data, particularly in June collections (Supplementary Fig. S2A,B). We therefore removed the three June samples from differential expression analysis. We then grouped the remaining collections into two groups based on hierarchical clustering, which suggested overall similarities among samples (Supplementary Fig. S2B). One group consisted of May, July, August, and September collections, and named ‘09MS’, and the other group included October collections and was named ‘09 O’.

### Assignment of 2015 and 2016 collections into four developmental states

**Figure 4:**
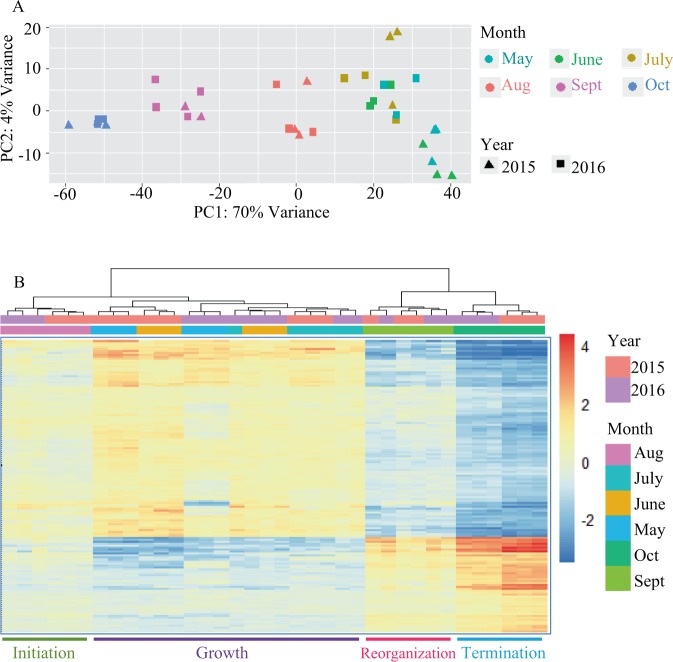
Clustering of 2015 and 2016 samples into four developmental states based on PCA and hierarchical clustering. (**A**) PCA plot of 2015 and 2016 RNA-Seq data. (**B**) Hierarchical clustering of monthly collections from 2015 and 2016, which was based on the relative expression of the top 150 DEGs (with lowest FDR values) identified in the pair-wise comparison between May 2015 and October 2015. In both PCA plot and the DEG-based heatmap, we recognized four states and named them: active growth, senescence initiation, reorganization, and senescence termination.

PCA plot of 2015 and 2016 data indicated that collections from these two years formed four chronological groups: May, June, and July collections from both years clustered together and formed one group, meanwhile August, September, and October collections over both years each formed an individual month-based cluster (Fig. 4A). Comparison of each monthly collection in 2015 to the same month in 2016 revealed that 395 (1.1%) to 6,233 (18.0%) of the expressed 34,623 genes were differentially expressed (FDR < 0.05, LFC cutoff = 0) between years (Supplementary Fig. S3). Within each year, 224 (0.7%) to 15,798 (45.6%) of the expressed genes were differentially expressed (FDR < 0.05, LFC cutoff = 0) among months (Supplementary Table S5; Supplementary Table S6). Consistent with PCA-generated groups, clustering of samples based on the 150 most highly differentially expressed genes identified in the comparison between May 2015 and October 2015 produced four same chronological groups (Fig. 4B). We classified these groups as ‘active growth’, ‘senescence initiation’, ‘reorganization’, and ‘senescence termination’, respectively, according to the leaf senescence phases defined by Munné-Bosch (see Introduction and Discussion).

### Identification of DEGs

Comparison of the two 2009 groups, ‘09MS’ and ‘09 O’, identified 10,375 DEGs (FDR < 0.05, LFC cutoff = 0), including 6,093 downregulated and 4,282 upregulated genes as leaves senesced (Supplementary Table S7).

Comparison of every two chronologically successive developmental states from 2015 and 2016 showed that a total of 14,415 (41.6%) of the expressed 34,623 genes were differentially expressed (FDR < 0.05, LFC cutoff = 0) in three developmental transitions, with 7,198, 7,091, and 5,698 DEGs associated with the transitions from growth to senescence initiation, from reorganization to termination, and from senescence initiation to reorganization, respectively (Supplementary Table S7). In each transition, the number of downregulated genes was slightly larger (*i.e*., 172 to 943 more) than the number of upregulated genes.

Combining 2009- and 2015/2016-based results, there were a total of 17,974 DEGs associated seasonal leaf growth and senescence. Among these DEGs, 3,939 (21.9%, including 2,212 downregulated and 1,727 upregulated) genes were only identified in 2009, and 8,988 (50.0%, including 4,512 downregulated and 4,476 upregulated) genes were specifically identified in 2015 and 2016. Meanwhile, 6,436 (35.8%, including 3,881 downregulated genes and 2,555 upregulated) genes were shared among years (Supplementary Fig. S4). In addition, 1,116 (6.2%) of the DEGs had no *Arabidopsis* match, and therefore were considered as novel (Supplementary Table S7).

### Correlations between RNA-Seq and qRT-PCR expression levels

**Figure 5:**
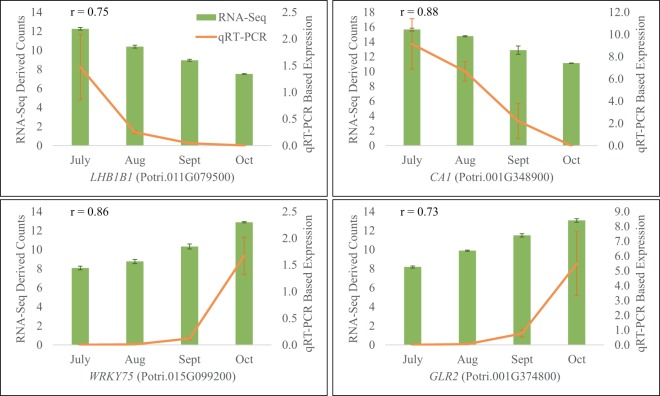
qRT-PCR validation of RNA-Seq data. Green bars represent DESeq. 2-normalized read counts, orange lines represent qRT-PCR determined relative transcript levels, error bars indicate standard errors (SEs) over three biological replicates, r values indicate Pearson correlation coefficients.

To validate RNA-Seq reflected transcript level, we performed qRT-PCR and determined the expression levels of four selected DEGs, *PtLHB1B1*, *PtCA1*, *PtWRKY75*, and *PtGLR2*, relative to the reference gene *PtEF1-beta*, in July, August, September, and October of 2015. Consistent with RNA-Seq data, qRT-PCR result showed that the expression of *PtLHB1B1* and *PtCA1* decreased, while the expression of *PtWRKY75* and *PtGLR2* kept increasing, during the onset and progression of senescence (Fig. 5). Pearson correlation coefficients ranged from 0.73 to 0.88 for each gene. Regression analysis of tree means indicated a quadratic relationship between the RNA-Seq data and qRT-PCT data (*p* < 0.001 for the quadratic term) fit our data better than a linear relationship (Supplementary Fig. 5).

### GO terms enriched in DEGs among leaf developmental states

**Figure 6:**
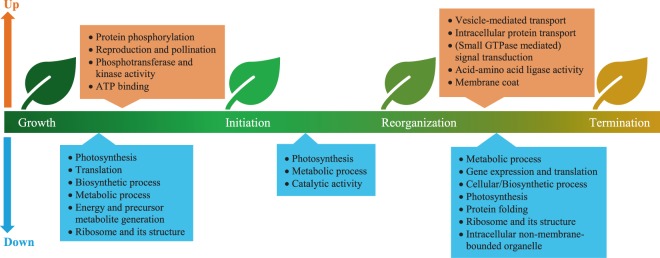
Categories of GO terms most significantly (*P* < 0.0001) enriched using upregulated and downregulated genes identified during leaf senescence. Detailed GO term IDs and annotations can be found in Supplementary Table S8.

To reveal functional categories associated with leaf senescence, we performed GO enrichment using the 14,415 DEGs identified from 2015 and 2016 data. A total of 279 GO terms were significantly (*p* < 0.05) enriched during the three developmental transitions (Supplementary Table S8). The transition from initiation to reorganization had fewest number of significant (*p* < 0.05) GO terms, with only 26 terms enriched, while each of the other two transitions had 166 terms. A total of 115 GO terms were specifically enriched in downregulated genes. Upon the initiation of senescence, genes encoding ribosome, chloroplasts, and other organelles were downregulated, indicated by the enrichment of cellular component terms, such as GO:0005840 ribosome, GO:0009579 thylakoid, GO:0044436 thylakoid part, and GO:0034357 photosynthetic membrane (Supplementary Table S8). Biological process terms related to photosynthesis (for example, GO:0009765, GO:0015979, and GO:0019684), metabolic activity (for example, GO:0008152), and energy generation (for example, GO:0006091), were also enriched in downregulated genes. Many of these cellular component and biological process terms were constantly downregulated throughout the process of senescence. In addition, as senescence progressed from initiation to reorganization, the expression levels of genes regulating catalytic activity (GO:0003824) were declined (Supplementary Table S8; Fig. 6).

Concurrently, we found 162 GO terms enriched in upregulated genes. Several biological process terms linked to protein phosphorylation (for example, GO:0006468, GO:0006796, and GO:0006793) were enriched during senescence initiation and reorganization (Supplementary Table S8), confirming the presence of post-translational level regulation of leaf senescence. Several GO terms related to reproduction and pollination (GO:0009875 pollen-pistil interaction, GO:0048544 recognition of pollen, GO:0009856 pollination, GO:0000003 reproduction, and GO:0022414 reproductive process) were enriched in the transition from growth to initiation (Fig. 6; Supplementary Table S8). The 48 genes enriched in these GO categories were homologous to 11 *Arabidopsis* gene models; nearly all these genes were associated with kinase activities (Supplementary Table S9). In the transition from reorganization to termination, active relocation occurred, as multiple GO terms, such as GO:0016192 vesicle-mediated transport, GO:0051641 cellular localization, GO:0051649 establishment of localization in cell, and GO:0008104 protein localization, were enriched (Fig. 6; Supplementary Table S8).

### Activities of TFs and AS factors during leaf senescence

Among the 14,415 DEGs obtained from 2015 and 2016 data, we identified a total of 881 differentially expressed TF genes from 54 TF families (Table 1). We found that several TF families that are widely known to regulate leaf senescence, such as NAC, WRKY, MYB, C2H2, and bZIP, were among the largest families that were active during our study period. Particularly, the majority of TF genes in the NAC and WRKY families were upregulated in more than one of the three developmental transitions (Supplementary Fig. S6). We also found that bHLH, ERF, and MYB_related TF families were overrepresented in genes both downregulated and upregulated (Table 1; Supplementary Fig. S6).

**Table 1:** Number of differentially expressed TFs during leaf development.

TF Family (Total No. in *P. trichocarpa*)	No. (%) of Differentially Expressed TF Genes	
bHLH (202)	77	(38.12)
MYB (213)	67	(31.46)
ERF (175)	66	(37.71)
MYB_related (101)	55	(54.46)
NAC (170)	51	(30.00)
WRKY (102)	51	(50.00)
C2H2 (142)	46	(32.39)
bZIP (96)	42	(43.75)
G2-like (66)	33	(50.00)
FAR1 (51)	30	(58.82)
GRAS (106)	29	(27.36)
HD-ZIP (63)	27	(42.86)
C3H (62)	23	(37.10)
ARF (35)	22	(62.86)
Trihelix (59)	19	(32.20)
GATA (39)	17	(43.59)
B3 (110)	16	(14.55)
Dof (45)	16	(35.56)
SBP (30)	16	(53.33)
TALE (34)	15	(44.12)
TCP (37)	14	(37.84)
MIKC-MADS (51)	12	(23.53)
ARR-B (17)	9	(52.94)
CO-like (17)	9	(52.94)
DBB (19)	9	(47.37)
HB-other (16)	8	(50.00)
HSF (30)	8	(26.67)
Nin-like (20)	8	(40.00)
AP2 (31)	6	(19.35)
E2F/DP (9)	6	(66.67)
GRF (19)	6	(31.58)
NF-YA (13)	6	(46.15)
NF-YB (21)	6	(28.57)
BES1 (14)	5	(35.71)
LBD (58)	5	(8.62)
M-type_MADS (54)	4	(7.41)
NF-YC (18)	4	(22.22)
YABBY (12)	4	(33.33)
ZF-HD (21)	4	(19.05)
BBR-BPC (16)	3	(18.75)
CPP (12)	3	(25.00)
EIL (7)	3	(42.86)
LSD (5)	3	(60.00)
NF-X1 (3)	3	(100.00)
CAMTA (7)	2	(28.57)
GeBP (6)	2	(33.33)
HB-PHD (4)	2	(50.00)
VOZ (4)	2	(50.00)
WOX (18)	2	(11.11)
HRT-like (1)	1	(100.00)
S1Fa-like (2)	1	(50.00)
SRS (10)	1	(10.00)
STAT (2)	1	(50.00)
Whirly (3)	1	(33.33)
**Total**	**881**	

**Figure 7:**
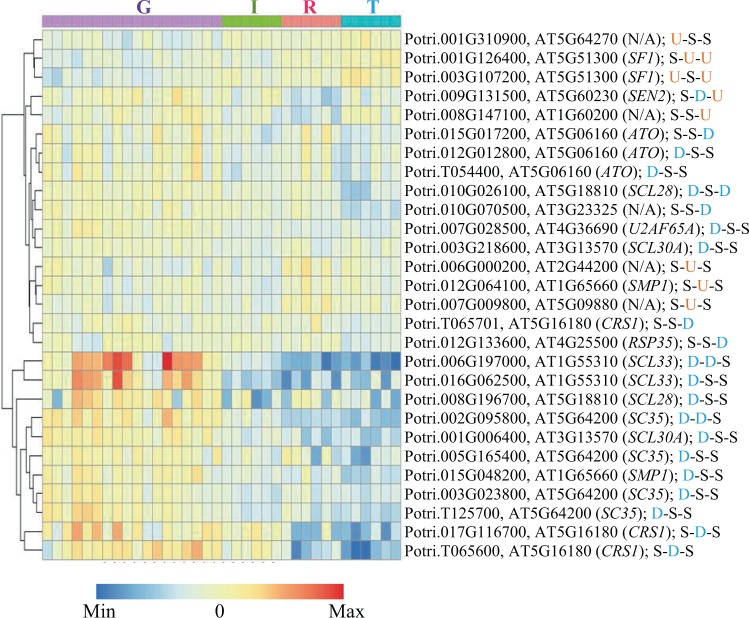
Expression profile of differentially expressed alternative splicing (AS) factors among developmental states. G = active growth; I = senescence initiation, R = reorganization, and T = senescence termination. For each gene, its *Populus* gene ID, corresponding *Arabidopsis* homolog (ID and name), and changes in expression, are shown. D = downregulated; U = upregulated; S = same (*i.e*., no significant changes).

Similarly, we identified 28 AS factor encoding genes from the 2015/2016-based DEGs. The majority of these genes were downregulated upon senescence initiation. Only eight genes, including two *SPLICING FACTOR 1* (*SF1*) homologs and one *SWELLMAP 1* (*SMP1*) homolog, were upregulated (Fig. 7).

### Clustering of DEGs by expression pattern and identification of sequence motifs associated with leaf senescence

**Figure 8:**
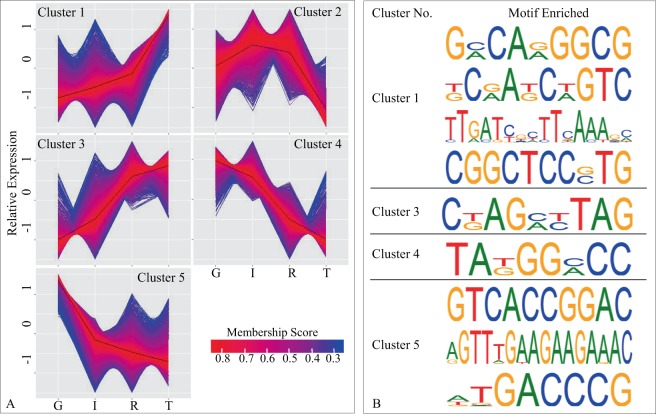
Identification of gene expression patterns and enrichment of motif analysis. (**A**) Five distinct expression patterns identified among the four recognized developmental states, active growth (G), senescence initiation (I), reorganization (R), and senescence termination (T). (**B**) The most high-confidence sequence motifs enriched using genes with membership scores higher than 0.80 in each cluster.

Using fuzzy C-means clustering algorithm, the 14,415 2015/2016-based DEGs were grouped into five distinct clusters (Supplementary Fig. S7; Supplementary Table S10), with each cluster containing 1,770 to 3,548 genes (Supplementary Table S11; Supplementary Table S12). Genes in clusters 1 and 3 showed increased expression during leaf senescence, while genes in clusters 4 and 5 showed reduced expression in general. Genes in Cluster 2 were upregulated from growth to initiation but downregulated in later developmental states (Fig. 8A).

To identify overrepresented sequence motifs associated with downregulated and upregulated genes during senescence, we performed motif enrichment using genes with membership scores higher than 0.80 in clusters 1, 3, 4, and 5, respectively. For each cluster, we identified 34 motifs using MEME and HOMER (Supplementary Table S13), and one to four high confidence motifs after filtering with the Ensemble algorithm (Fig. 8B). Comparison against *Arabidopsis* motif database *Arabidopsis*DAP (v1) showed that seven of the nine high-confidence motifs matched to known motifs associated with various TFs, such as bZIP, TCP, NAC, Trihelix, and bHLH (Supplementary Table S14).

### DEGs shared with age-dependent senescence, seasonal bud dormancy, and cold acclimation

**Figure 9:**
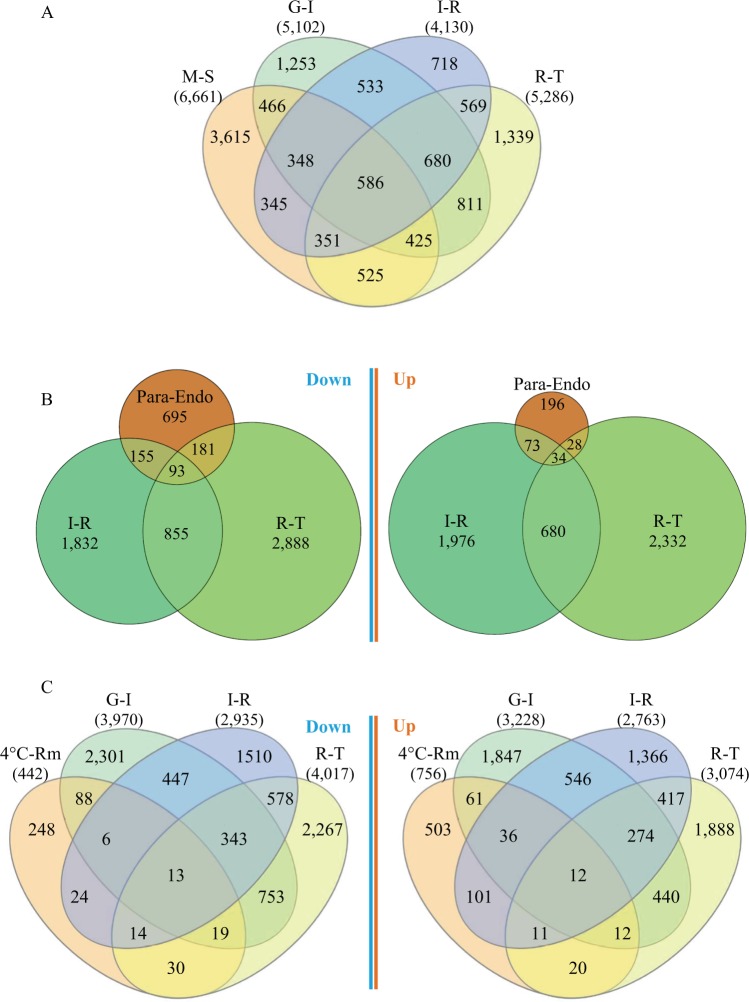
Number of DEGs that are in common with or distinct from age-dependent senescence in *Arabidopsis* (**A**) and vegetative bud dormancy (**B**) and cold acclimation (**C**) in *P. trichocarpa*, respectively. The *Arabidopsis* senescence data was generated by Woo *et al*.^7^, the *Populus* dormancy data was generated by Howe *et al*.^32^, and the *Populus* cold acclimation data was generated by Chen *et al*.^33^. G-I = active growth to senescence initiation; I-R = senescence imitation to reorganization; R-T = reorganization to senescence termination; M-S = mature to senescence; Para-Endo = paradormancy (August) to endodormancy (November and December); 4 °C-Rm = cold treatment at 4 °C *vs*. control at room temperature. The four-way Venn diagrams in panels (A) and (C) were created using the web-based tool InteractiVenn, and the weighted three-way Venn diagrams in panel (B) were created using the package ‘eulerr’ in R (version 3.5.2, 2018; see Methods for details).

To relate genes identified in this study to SAGs previously discovered, we compared 2015/2016-based DEGs, representing 8,949 *Arabidopsis* gene models, against the 6,661 *Arabidopsis* genes reported by Woo *et al*.^7^ in studying age-dependent senescence. A total of 3,046 *Arabidopsis* gene models were shared between seasonal senescence in *Populus* and age-dependent senescence in *Arabidopsis*; meanwhile, 3,615 and 5,903 *Arabidopsis* gene models were uniquely identified in age-dependent senescence and seasonal senescence, respectively (Fig. 9A; Supplementary Table S15). We did not distinguish upregulated and downregulated genes in the comparison because this information was not reported by Woo *et al*.^7^.

To determine the level of similarity in genetic control of seasonal leaf senescence and bud dormancy, we compared the DEGs identified in the transitions from senescence initiation to reorganization and from reorganization to termination to the 1,455 *Populus* DAGs (including 1,124 downregulated genes and 331 upregulated genes) identified by Howe *et al*.^32^ in the transition from paradormancy (August) to endodormancy (November and December). We found a total of 564 genes (including 429 downregulated genes and 135 upregulated genes) that were in common (Fig. 9B; Supplementary Table S15). These genes, in particular, the downregulated ones, were enriched in 28 GO terms (Supplementary Table S16), among which six GO terms (GO:0015979, GO:0009765, GO:0019684, GO:0034357, GO:0009521, and GO:0009579), all related to photosynthesis, were most significantly (*p* < 0.001) enriched.

Similarly, to explore genes in common with those responding to cold acclimation, we compared our DEGs with the 1,198 *Populus* CAGs in response to low temperature treatment at 4 °C reported by Chen *et al*.^33^. In total, there were 447 genes (including 194 downregulated genes and 253 upregulated genes) shared by both senescence and cold acclimation (Fig. 9C; Supplementary Table S15). We obtained 29 GO terms, for example, those related to biosynthesis and metabolic process, enriched in the shared upregulated genes (Supplementary Table S16).

## Discussion

Here we describe a time-course analysis of transcriptome changes during *Populus* leaf development and senescence over three years. We used RNA-Seq, a revolutionary tool for transcriptome profiling^36^, and *P. trichocarpa* Nisqually 1, the reference genome^20^. Due to in depth library sequencing, we were able to obtain numerous low abundance transcripts; we detected expression of 36,007 genes during the collection period. This number nearly tripled the number of genes examined in the microarray-based *Populus* senescence transcriptome study conducted by Andersson *et al*.^15^. In differential expression analysis, we set FDR to 0.05 and LFC cutoff to zero to maximize the chance of discovering novel SAGs. We were able to identify 17,974 DEGs, including 1,116 genes without known *Arabidopsis* matches and those represented 5,903 *Arabidopsis* genes that were not previously reported in age-dependent leaf senescence transcriptomes^7^.

The variation in gene expression and its connection to physiological progression of senescence that we observed in *Populus* largely concur with what have been reported in *Arabidopsis* and other plant species. For example, gene expression during active growth varied widely among samples, much more than did samples undergoing senescence, which is evident from our PCA plots (Fig. 4A) and differential expression analysis (Supplementary Fig. S3) of the RNA-Seq data. A similar observation that growth is under less tight genetic control compared with senescence was reported in *Arabidopsis*^7^. We observed the first set of considerable changes in gene expression in August (as reflected by the color change in Fig. 4B), suggesting the initiation of senescence according to Munné-Bosch’s model^3^. Although GO analysis indicated that genes related to thylakoid and its structure started to be downregulated in the transition from growth to initiation (Supplementary Table S8), physiologically we did not observe significant decrease in chlorophyll content until September (Fig. 3A,C), which corresponds with the start of the reorganization phase^3^. As part of the final stages of cell death and leaf senescence, the plasma membrane is disrupted resulting in leaf electrolyte leakage, which can be quantified by conductance – which has been proposed to be a reliable indicator of the terminal phase of senescence^1,17^. October collections from both years showed high levels of conductance, indicating massive membrane deterioration and the final stage of senescence. Interestingly, May collections also showed high levels of conductance. This phenomenon might be due to the acquisition of ammonium and other nutrient during active growth^37^ but not loss of plasma membrane integrity. Munné-Bosch’s model^3^ suggests that nutrient translocation occurs at the late stages of reorganization. We found active relocation, as suggested by GO enrichment (Fig. 6), in October but not September. Nonetheless, we classified October as in the termination phase, as both increased leaf electrolyte leakage and overrepresentation of transport-related GO terms indicated the final stage of leaf senescence were underway.

We found that the 2009 data was poorly correlated with the 2015 or 2016 data. This might be due to a number of factors, including a change in the trees sampled in most recent two years and the use of a different sequencing platform. In 2015 and 2016 single-end 100 pb reads were produced on Illumina Hiseq. 3000, which is a superior, higher depth (nearly 7.5 fold more reads per lane) platform than Illumina GAIIx^38,39^ that was used for producing paired-end 50 bp reads from 2009 samples. Given this difference, we were surprised to find that 3,939 DEGs were identified in 2009 but not in 2015/2016. It is possible that the classification of the 2009 samples into two general groups enabled the identification of DEGs that had subtle changes among the four chronological groups used for analysis of the 2015 and 2016 samples.

During our sampling period, seasonal bud dormancy and cold acclimation seemingly occurred simultaneously with leaf senescence, however we found low percentage (39% or less) of DEGs shared between senescence and dormancy, and between senescence and cold acclimation. These results agree with previous findings in silver birch (*Betula pendula*) that seasonal leaf senescence, bud dormancy, and cold acclimation are three relatively independent, though commonly co-occurring, processes^40^.

The enrichment of GOs that have usually been found to be associated with senescence, including photosynthesis, metabolic process, kinase activity, and transport, suggest that seasonal leaf senescence have a lot of in common with age-dependent senescence. In addition, we found that GOs related to negative regulation of pollination were upregulated in the transition from growth to senescence initiation. Examination of the associated genes showed that it was due to the presence of 48 genes with general functions related to protein kinase activity. In fact, a number of protein kinases have been found to participate in leaf senescence^41,42^. Therefore, the overrepresentation of these reproductive-related GOs is unlikely to indicate a direct association of pollination-specific transcription and the transition from growth to initiation of senescence.

Previous studies on transcriptome profiling of seasonal leaf senescence in perennials did not specifically identify TF genes^15,16^. Our identification of 881 differentially expressed TF genes from 54 different TF families confirmed the crucial role of TFs in leaf senescence in perennials as in annual plants. We found that a number of TF genes in the NAC, WRKY, and MYB families were upregulated during senescence, agreeing their roles as positive regulators of senescence reported previously^8,43^. Interestingly, the bHLH family, which had the largest number of genes differentially expressed in our study, was not significantly enriched in DEGs involved in leaf senescence in several species, such as *Arabidopsis*^6^ and wheat^9^. In cotton, a number of bHLH TF genes were found to be upregulated during senescence^11^. In our study, however, about two thirds of differentially expressed bHLH genes were downregulated during leaf development and senescence (Supplementary Fig. S6). The functions of bHLH TFs in *Populus* largely remain uncharacterized. In *Arabidopsis*, there are 162 bHLH TF genes forming 21 subfamilies^44,45^; their roles have been shown to include regulation of fruit dehiscence, anther and epidermal cell development, hormone signaling, and stress responses^46,47^. One sub-group, VII (a + b), contains members, such as PIF1, PIF3, PIF4, and PIF5, reacting to light signaling^47^. Upon exposure to light, followed by phytochrome binding, these PIFs are degraded and photomorphogenesis is triggered^48^. Furthermore, the circadian clock elements *PIF4* and *PIF5* have been upregulated and work together with other genes to activate *ORE1* and other downstream SAGs in dark-induced senescence^49–51^. In our study, however, we found that one *PtPIF4* (*Potri.005G207200*) were downregulated in all three developmental transitions, while the other *PtPIF4* (*Potri.002G055400*) were downregulated in the transition from growth to initiation, but upregulated in the transition from reorganization to termination. Despite the distinct changes of *PIF4* in seasonal senescence in trees and dark-induced senescence in annual species, the overrepresentation of bHLH TFs in the DEGs identified in our study suggests the role of these TFs in seasonal leaf senescence in response to shortening day length.

The ERF family was also among the most active TF families identified in our study, with most of the TF genes downregulated (Supplementary Fig. S6). This observation is consistent with previous findings that a number of ERF TFs are negative regulators of leaf senescence^52^. It also suggests potential cross-talk between seasonal leaf senescence and ethylene signaling pathways, as the ERF TFs bind to ethylene-responsive elements.

Despite that AS has been shown to participate in regulating plant development and defense^53,54^, its role in leaf senescence has not been well characterized^8^. In this study, we were able to recognize 28 AS factor encoding genes that were differentially expressed during leaf senescence, with most of them were downregulated (Fig. 7). There were two AS factor genes, homologues to *Arabidopsis SF1*, showing increased expression level as leaves senesced. In *Arabidopsis*, *sf1* mutants have been reported to grow shorter, have smaller leaves, flower early, and have abnormal phyllotaxy in the inflorescence^55^, suggesting a broad role for *SF1* in regulating plant development, including reproduction-associated leaf senescence.

We were able to enrich dozens of sequence motifs. Some of these motifs have not been reported before, such as “GMCARGGCG” and “CKAGMYTAG” (Supplementary Table S14). Some motifs have been previously found in SAGs or stress-response genes. For example, the motif ‘KCRAKCWGTC,’ overrepresented in genes upregulated over our study period (Fig. 8), is present in the promoter regions of *Arabidopsis* genes *bZIP69* and *bZIP18* (Supplementary Table S14) – two genes that were found to be highly expressed at the leaf senescence stage^56^. Several motifs enriched with genes downregulated during senescence, such as ‘TAKGGMCC’ and ‘AGTTTGAAGAAGAAAC,’ were previously identified from *TCP* (*e.g*., *TCP17* and *TCP24*) and *NAC* genes (*e.g*., *ANAC075* and *ANAC071*), respectively, which have been found to respond to senescence or abiotic stresses^57–60^.

The results of this study could have applications in biotechnology. For example, the identification of sequence motifs enriched in upregulated genes in this study could inform efforts to engineer the biosynthesis of coproducts, such as biopolymers or proteins, in leaves in association with the onset of senescence (to reduce the drain of production during active plant growth and photosynthesis). Polyhydroxybutyrate (PHB), a source for biodegradable and stainable plastics, can be synthesized in leaves but has been shown to have negative impacts on plant growth^61,62^. The promoters of genes that were only upregulated during senescence could potentially be used to control PHB gene expression to minimize the negative effects of biosynthesis during growing season.

## Supplementary information

Supplementary information.

Supplementary information2.

Supplementary information3.

Supplementary information4.

Supplementary information5.

Supplementary information6.

Supplementary information7.

Supplementary information8.
