# Supplementary material for: Extensive transcriptome changes during seasonal leaf senescence in field-grown black cottonwood (Populus trichocarpa Nisqually-1)
Source: Sci Rep. 2020 Apr 20;10:6581. doi: 10.1038/s41598-020-63372-2 (PMC7170949; doi:10.1038/s41598-020-63372-2)
Supplement: Supplementary file 1 — Supplementary information. [file 41598_2020_63372_MOESM1_ESM.pdf]

Supplementary information

Extensive transcriptome changes during seasonal leaf senescence in field-grown black cottonwood (*Populus trichocarpa* Nisqually-1)

Haiwei Lu, Michael Gordon, Vindhya Amarasinghe, & Steven H. Strauss*

Department of Forest Ecosystems and Society, Oregon State University, Corvallis, Oregon, USA. Correspondence and requests for materials should be addressed to S.H.S. (email: Steve.Strauss@oregonstate.edu)

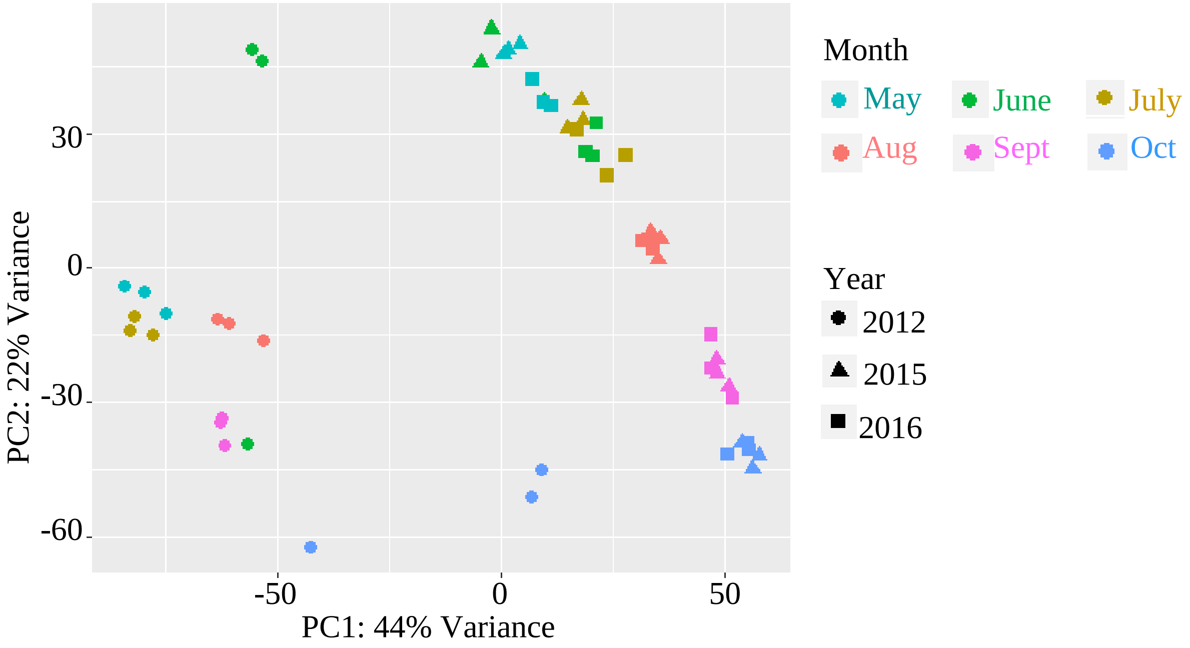

Supplementary Figure S1. Principal component analysis of three years' RNA-Seq data revealed large variance between 2009 and 2015/2016 collections.

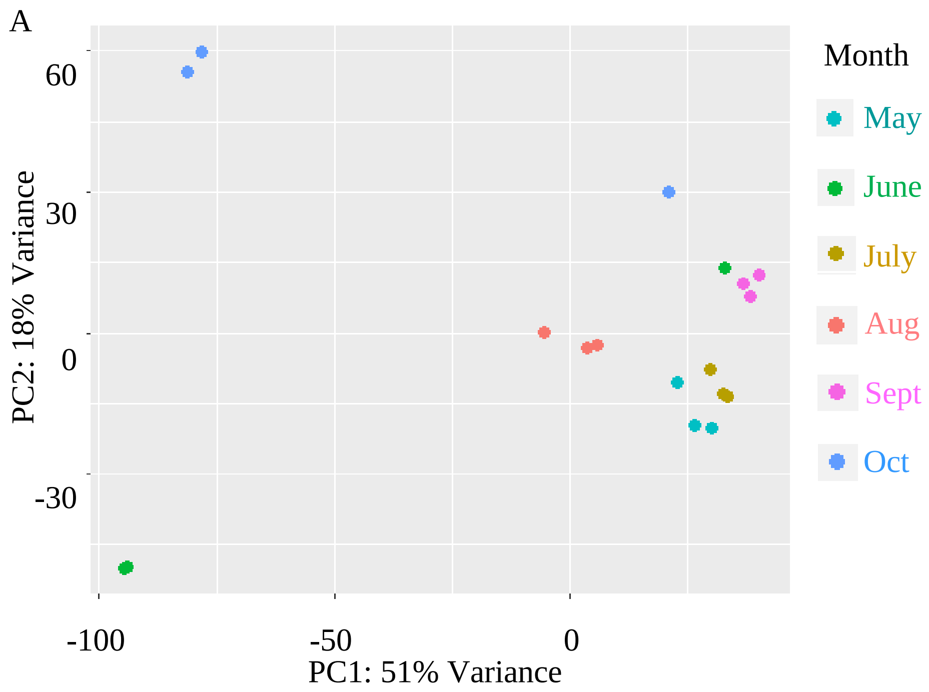

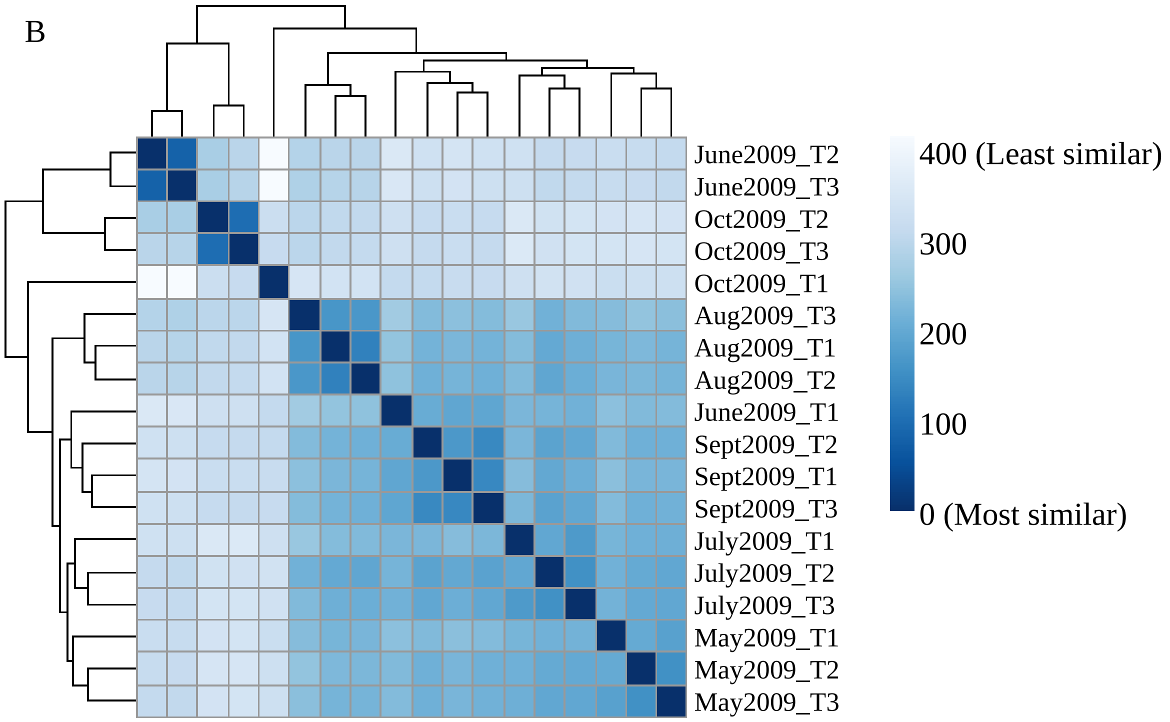

Supplementary Figure S2. Examination of variation among 2009 RNA-Seq data. (A) PCA plot revealed large variations within both June collections and October collections. (B) Heatmap of sample-to-sample distances, where similarities between samples were reflected by hierarchical clustering and darkness of the color, confirmed large variations within the June collections.

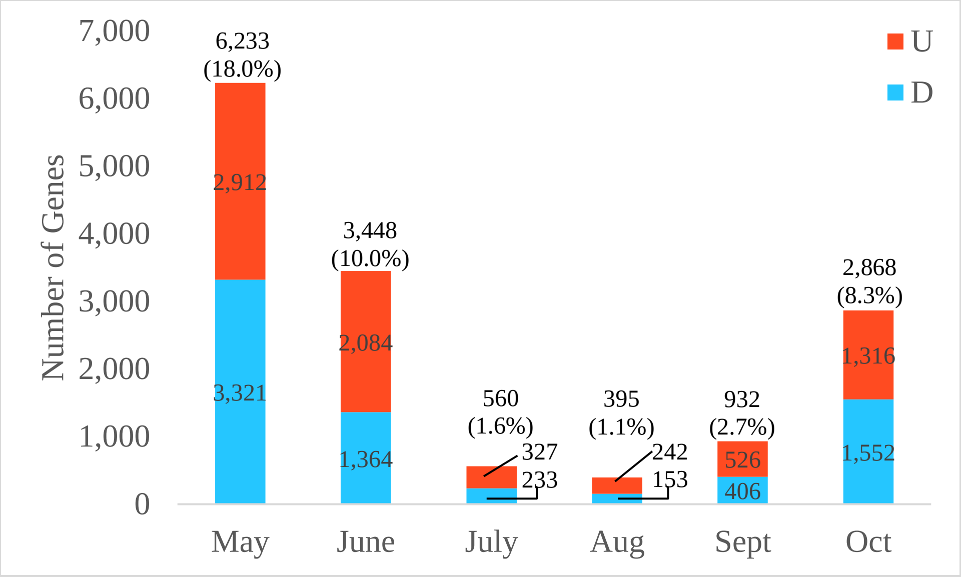

Supplementary Figure S3. Number of DEGs (FDR < 0.05, LFC cutoff = 0) from comparing the collection in 2015 to the same month in 2016. Orange (upper) bars and blue (lower) bars indicate upregulated (U) and downregulated (D) genes, respectively, in 2016, compared with 2015. Total number of gene models analyzed = 34,623.

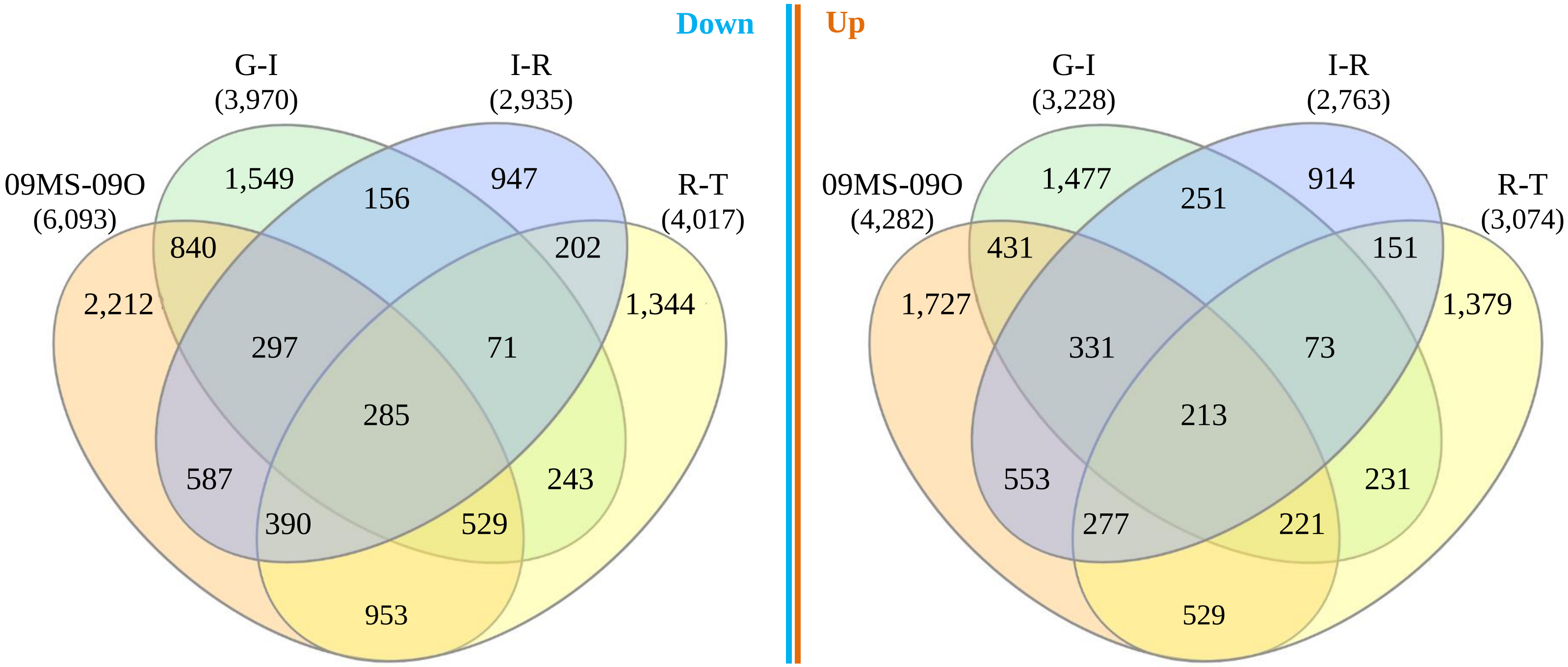

Supplementary Figure S4. Comparison of numbers of DEGs (FDR < 0.05, LFC cutoff = 0) that were shared or unique between 2009 and 2015 and 2016. 09MS= May, July, August, and September collections from 2009; 09O = Oct collections from 2009; G = growth (May, June, and July collections from 2015 and 2016), I = senescence initiation (Aug collections from 2015 and 2016), R = reorganization (September collections from 2015 and 2016); T = senescence termination (October collections from 2015 and 2016).

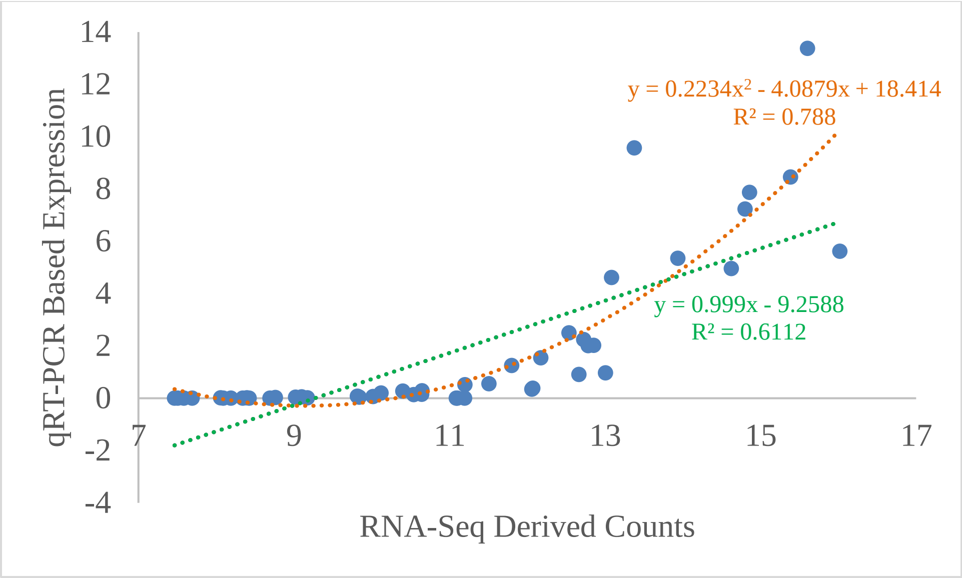

Supplementary Figure S5. Regression analysis of tree means of RNA-Seq data and qRT-PCR data for four analyzed genes (*PtLHB1B1*, *PtCA1*, *PtWRKY75*, and *PtGLR2*) at four collection timepoints (July, August, September, and October in 2015). The green line and orange line represent the fitted linear model and quadratic model, respectively.

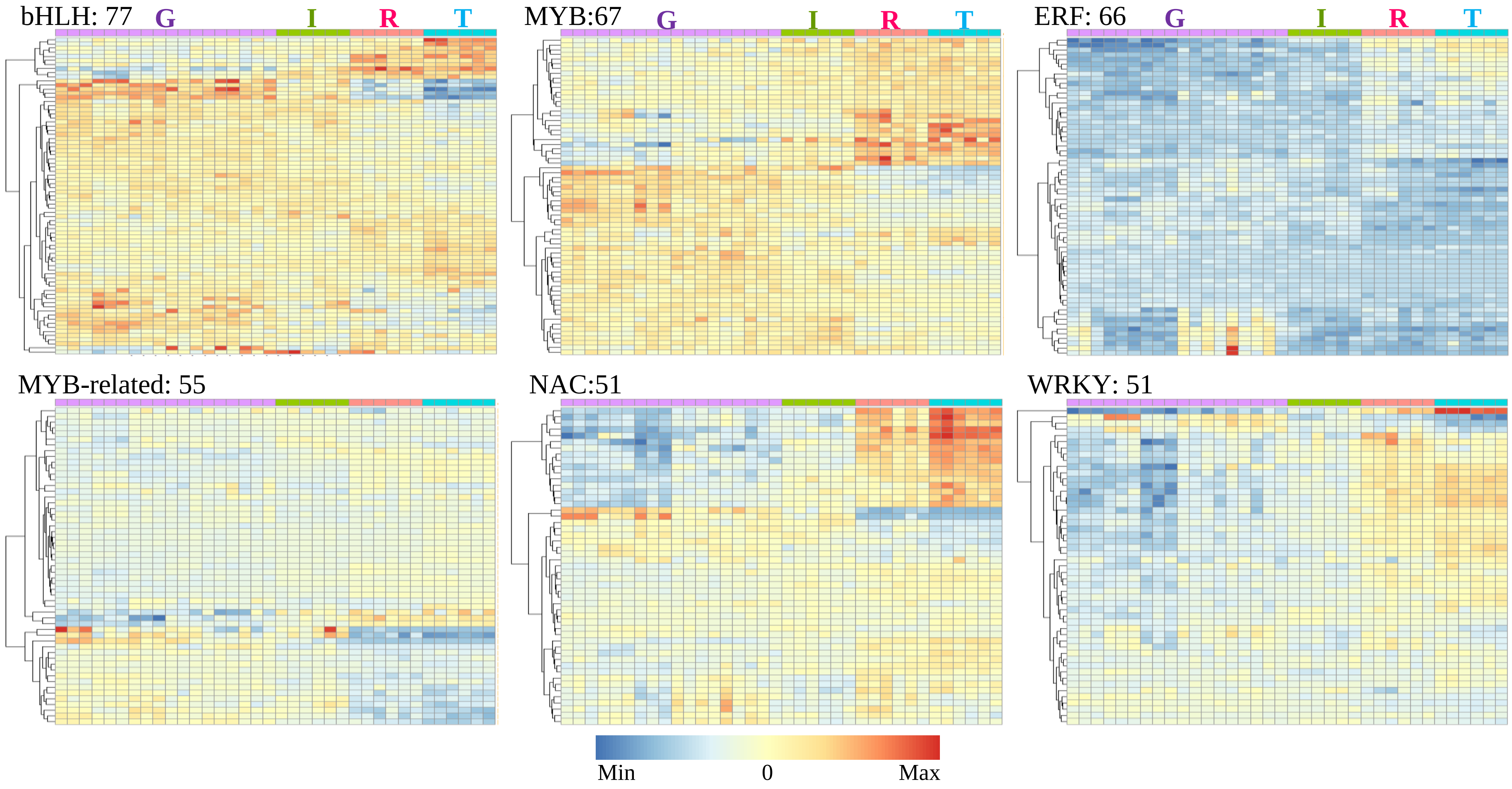

Supplementary Figure S6. Hierarchical cluster analysis of differentially expressed TFs from top six TF families over-represented in DEGs. Name of TF family and number of differentially expressed TF are labelled on top of each heatmap. G = active growth; I = senescence initiation, R = reorganization, and T = senescence termination.

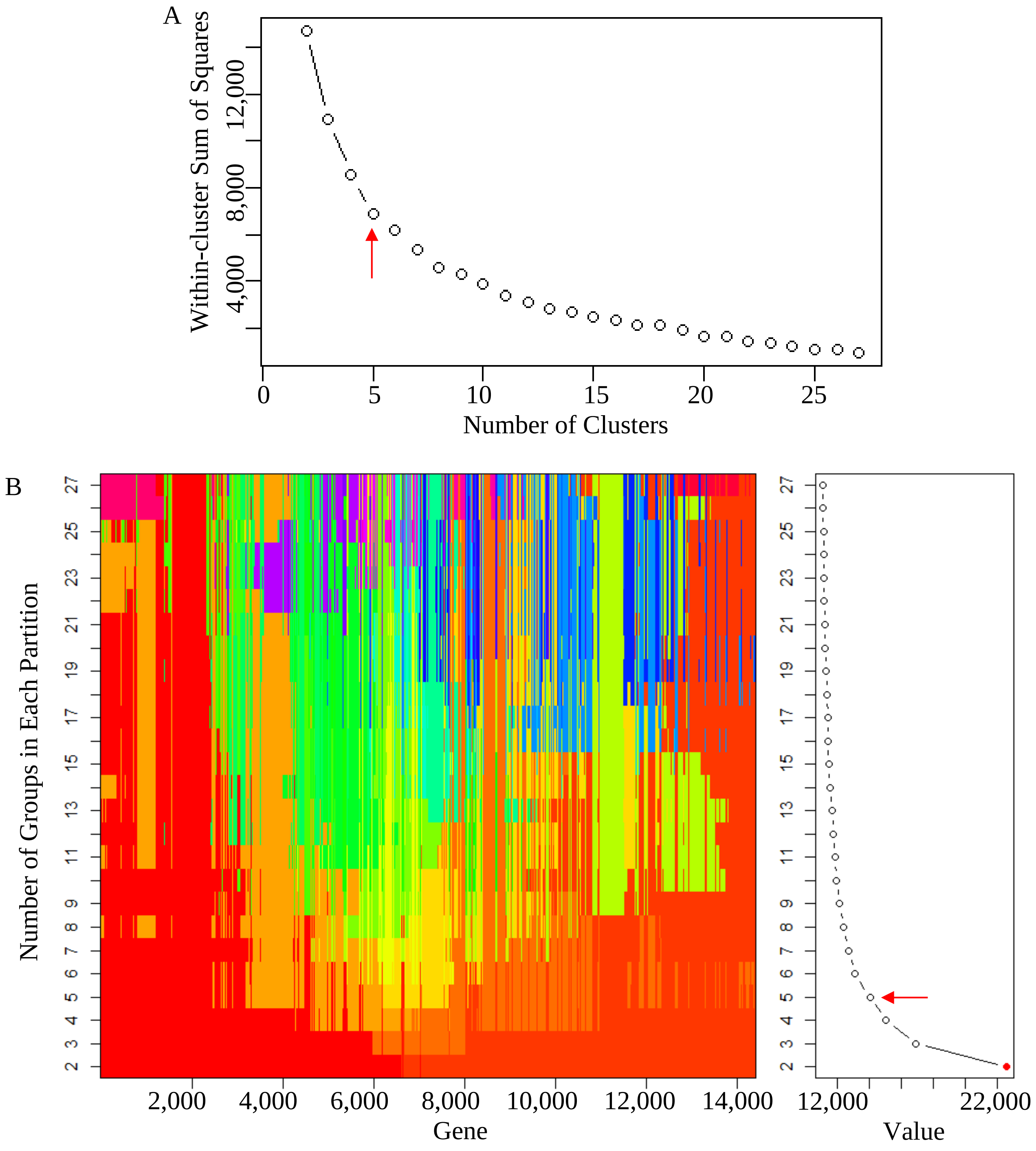

Supplementary Figure S7. Determination of number of clusters. (a) Plot of within-cluster sum of squares (WCSS) against number of clusters. (b) C-means partitions comparison (left panel) and Calinski-Harabasz index with values (right panel). Red arrows indicate the values of WCSS and Calinski-Harabasz index for four clusters, which was the selected number of clusters.

Supplementary Table S1 Primers used in qRT-PCR. Amplification efficiency (E) and correlation coefficient R^2 from standard curve are included.

**Gene Name**	**Gene ID**	**Sequence of Primer (5' - 3')**		**E (%)**	**R^2**
		**Forward**	**Reverse**		
LHB1B1	Potri.011G079500	TAAATACCTGGGTCCTTTCTCCG	CACACAGCCTCACCGAATTTG	87.8	0.998
CA1	Potri.001G348900	CCTCTGTTTCTCCTCCTACCCTTA	GCCTCGTTGTAGTCCTTTCCC	89.4	0.996
WRKY75	Potri.015G099200	TCAAGAGCAGCAAGTTTCCAAG	GTCACAACAATCCCTTCATCTTTAG	89.7	0.980
GLR2	Potri.001G374800	AACTCCCAGTGCCAAGATATTC	CGGAAACGCAAATCCAAACC	101.0	0.980
EF1-beta	Potri.009G018600	AACCTGGTCGTGATTTCCCT	ATCACCAGCAGCCTCCTTG	96.0	0.995

Supplementary Table S2 Chlorophyll content index (CCI) and electrolyte leakage index (ELI) of leaf samples collected in 2015 and 2016. Letters indicate statistical significance in comparison among months using ANOVA ($p < 0.05$).

Month	CCI (2015)			ELI (2015)			CCI (2016)			ELI (2016)		
	**Ave**	**(±SE)**	**Group**	**Ave**	**(±SE)**	**Group**	**Ave**	**(±SE)**	**Group**	**Ave**	**(±SE)**	**Group**
May	35.35	(±0.50)	c	0.173	(±0.006)	bc	39.72	(±0.31)	b	0.183	(±0.003)	c
June	38.46	(±0.41)	d	0.144	(±0.003)	a	39.53	(±0.33)	b	0.146	(±0.003)	ab
July	36.11	(±0.39)	cd	0.149	(±0.003)	a	38.67	(±0.59)	b	0.151	(±0.004)	ab
Aug	35.24	(±0.39)	c	0.156	(±0.002)	ab	37.90	(±0.76)	b	0.141	(±0.003)	a
Sept	31.07	(±0.67)	b	0.179	(±0.004)	c	32.47	(±0.61)	a	0.163	(±0.003)	b
Oct	23.48	(±1.11)	a	0.191	(±0.008)	c	31.85	(±1.10)	a	0.188	(±0.009)	c

Supplementary Table S3 Summary of RNA-Seq data and mapping results from the HISAT2 pipeline.

Sample ID	No. of Total Reads	No. (%) of Reads Aligned to the Genome					
		Total		Singletons		Multiple Alignments	
2009_May_T1	14,520,199	6,824,038	(47.00)	6,440,037	(44.35)	384,001	(2.64)
2009_May_T2	24,279,468	14,417,279	(59.38)	13,466,872	(55.47)	950,407	(3.91)
2009_May_T3	19,839,959	10,540,220	(53.13)	9,873,859	(49.77)	666,361	(3.36)
2009_June_T1	44,513,826	24,832,680	(55.79)	23,316,084	(52.38)	1,516,596	(3.41)
2009_June_T2	65,076,413	19,367,433	(29.76)	17,139,117	(26.34)	2,228,316	(3.42)
2009_June_T3	59,826,566	41,100,852	(68.70)	36,563,402	(61.12)	4,537,450	(7.58)
2009_July_T1	39,626,491	21,852,230	(55.15)	20,666,670	(52.15)	1,185,560	(2.99)
2009_July_T2	33,815,032	20,413,901	(60.37)	19,327,143	(57.16)	1,086,758	(3.21)
2009_July_T3	37,568,045	22,895,111	(60.94)	21,658,411	(57.65)	1,236,700	(3.29)
2009_Aug_T1	12,394,663	7,104,219	(57.32)	6,721,991	(54.23)	382,228	(3.08)
2009_Aug_T2	19,388,292	9,550,976	(49.26)	9,035,216	(46.60)	515,760	(2.66)
2009_Aug_T3	18,765,531	6,045,587	(32.22)	5,670,737	(30.22)	374,850	(2.00)
2009_Sept_T1	32,098,088	22,097,826	(68.84)	20,772,479	(64.72)	1,325,347	(4.13)
2009_Sept_T2	31,704,374	21,977,599	(69.32)	20,737,904	(65.41)	1,239,695	(3.91)
2009_Sept_T3	30,795,614	21,011,933	(68.23)	19,796,477	(64.28)	1,215,456	(3.95)
2009_Oct_T1	28,065,529	12,072,485	(43.02)	11,097,135	(39.54)	975,350	(3.48)
2009_Oct_T2	64,688,503	15,277,822	(23.62)	13,843,986	(21.40)	1,433,836	(2.22)
2009_Oct_T3	58,792,079	39,165,232	(66.62)	35,869,958	(61.01)	3,295,274	(5.60)
2015_May_T1	39,028,092	27,888,662	(71.46)	22,609,980	(57.93)	5,278,682	(13.53)
2015_May_T2	37,618,748	26,251,025	(69.78)	22,413,106	(59.58)	3,837,919	(10.20)
2015_May_T3	41,864,782	27,650,663	(66.05)	20,314,760	(48.52)	7,335,903	(17.52)
2015_June_T1	38,383,412	17,807,531	(46.39)	16,145,327	(42.06)	1,662,204	(4.33)
2015_June_T2	36,034,085	23,978,088	(66.54)	21,472,638	(59.59)	2,505,450	(6.95)
2015_June_T3	40,129,485	22,027,015	(54.89)	20,115,478	(50.13)	1,911,537	(4.76)
2015_July_T1	35,998,575	24,853,715	(69.04)	22,489,369	(62.47)	2,364,346	(6.57)
2015_July_T2	35,816,142	22,244,782	(62.11)	19,801,104	(55.29)	2,443,678	(6.82)
2015_July_T3	38,873,070	28,479,578	(73.26)	25,672,341	(66.04)	2,807,237	(7.22)
2015_Aug_T1	44,057,801	30,042,542	(68.19)	25,723,478	(58.39)	4,319,064	(9.80)
2015_Aug_T2	35,787,635	18,966,324	(53.00)	16,468,303	(46.02)	2,498,021	(6.98)
2015_Aug_T3	35,853,042	19,030,108	(53.08)	17,393,980	(48.51)	1,636,128	(4.56)
2015_Sept_T1	43,618,559	29,951,318	(68.67)	26,360,131	(60.43)	3,591,187	(8.23)
2015_Sept_T2	36,097,415	22,640,046	(62.72)	18,304,820	(50.71)	4,335,226	(12.01)
2015_Sept_T3	39,873,012	30,333,682	(76.08)	27,022,196	(67.77)	3,311,486	(8.31)
2015_Oct_T1	48,464,438	32,054,327	(66.14)	26,104,222	(53.86)	5,950,105	(12.28)
2015_Oct_T2	37,174,028	23,676,235	(63.69)	17,746,854	(47.74)	5,929,381	(15.95)
2015_Oct_T3	37,428,947	18,530,556	(49.51)	16,013,466	(42.78)	2,517,090	(6.72)
2016_May_T1	45,063,842	30,750,956	(68.24)	28,099,664	(62.36)	2,651,292	(5.88)
2016_May_T2	40,719,864	30,604,908	(75.16)	28,376,455	(69.69)	2,228,453	(5.47)
2016_May_T3	36,905,845	26,253,475	(71.14)	24,263,463	(65.74)	1,990,012	(5.39)
2016_June_T1	40,347,881	23,476,652	(58.19)	21,592,650	(53.52)	1,884,002	(4.67)
2016_June_T2	34,019,874	18,230,475	(53.59)	16,759,721	(49.26)	1,470,754	(4.32)
2016_June_T3	43,456,828	27,921,987	(64.25)	26,049,163	(59.94)	1,872,824	(4.31)
2016_July_T1	41,609,097	30,710,589	(73.81)	27,742,782	(66.67)	2,967,807	(7.13)
2016_July_T2	39,097,800	22,427,788	(57.36)	17,264,364	(44.16)	5,163,424	(13.21)
2016_July_T3	40,315,148	28,209,676	(69.97)	24,938,798	(61.86)	3,270,878	(8.11)
2016_Aug_T1	39,008,336	26,647,504	(68.31)	21,110,739	(54.12)	5,536,765	(14.19)
2016_Aug_T2	46,381,838	30,952,623	(66.73)	25,213,638	(54.36)	5,738,985	(12.37)
2016_Aug_T3	42,925,471	18,296,141	(42.62)	15,583,289	(36.30)	2,712,852	(6.32)
2016_Sept_T1	34,782,308	21,619,829	(62.16)	19,555,925	(56.22)	2,063,904	(5.93)
2016_Sept_T2	44,279,522	34,089,344	(76.99)	29,991,551	(67.73)	4,097,793	(9.25)
2016_Sept_T3	33,957,595	21,402,180	(63.03)	16,693,598	(49.16)	4,708,582	(13.87)
2016_Oct_T1	35,202,786	24,877,046	(70.67)	21,960,129	(62.38)	2,916,917	(8.29)
2016_Oct_T2	35,163,735	18,148,158	(51.61)	14,921,418	(42.43)	3,226,740	(9.18)
2016_Oct_T3	33,293,602	21,651,088	(65.03)	20,182,296	(60.62)	1,468,792	(4.41)

Supplementary Table S4 Un-normalized read counts for each gene model generated using the HISAT2-StringTie pipeline.

The table was uploaded as a separate Excel file.

Supplementary Table S5 Number and percentage of DEGs (FDR < 0.05, LFC cutoff = 0) from pair-wise comparisons among months during the 2015 collections. D indicates down-regulated genes; U indicates up-regulated genes. Total number of gene models analyzed = 34,623.

**Month**	**June**		**July**		**Aug**		**Sept**		**Oct**	
	2,233	6.45%	3,747	10.82%	6,757	19.52%	11,542	33.34%	15,798	45.63%
**May**	D: 1,255	3.62%	D: 1,842	5.32%	D: 3,404	9.83%	D: 5,856	16.91%	D: 8,007	23.13%
	U: 978	2.82%	U: 1,905	5.50%	U: 3,353	9.68%	U: 5,686	16.42%	U: 7,791	22.50%
			2,830	8.17%	7,065	20.41%	13,160	38.01%	16,294	47.06%
**June**			D: 1,118	3.23%	D: 3,446	9.95%	D: 6,759	19.52%	D: 8,331	24.06%
			U: 1,712	4.94%	U: 3,619	10.45%	U: 6,401	18.49%	U: 7,963	23.00%
					4,860	14.04%	10,214	29.50%	14,788	42.71%
**July**					D: 2,502	7.23%	D: 5,262	15.20%	D: 7,680	22.18%
					U: 2,358	6.81%	U: 4,952	14.30%	U: 7,108	20.53%
							5,005	14.46%	11,981	34.60%
**Aug**							D: 2,472	7.14%	D: 6,256	18.07%
							U: 2,533	7.32%	U: 5,725	16.54%
									8,511	24.58%
**Sept**									D: 4,657	13.45%
									U: 3,854	11.13%

Supplementary Table S6 Number and percentage of DEGs (FDR < 0.05, LFC cutoff = 0) from pair-wise comparisons among months during the 2016 collections. D indicates down-regulated genes; U indicates up-regulated genes. Total number of gene models analyzed = 34,623.

Month	June		July		Aug		Sept		Oct	
May	5,582	16.12%	5,852	16.90%	8,932	25.80%	12,549	36.24%	15,059	43.49%
	D: 2,540	7.34%	D: 2,546	7.35%	D: 4,181	12.08%	D: 6,124	17.69%	D: 7,629	22.03%
	U: 3,042	8.79%	U: 3,306	9.55%	U: 4,751	13.72%	U: 6,425	18.56%	U: 7,430	21.46%
June			224	0.65%	1,963	5.67%	8,635	24.94%	12,789	36.94%
			D: 128	0.37%	D: 963	2.78%	D: 4,369	12.62%	D: 6,690	19.32%
			U: 96	0.28%	U: 1,000	2.89%	U: 4,266	12.32%	U: 6,099	17.62%
July					1,685	4.87%	8,613	24.88%	12,717	36.73%
					D: 854	2.47%	D: 4,506	13.01%	D: 6,864	19.82%
					U: 831	2.40%	U: 4,107	11.86%	U: 5,853	16.90%
Aug							4,689	13.54%	10,586	30.58%
							D: 2,497	7.21%	D: 5,721	16.52%
							U: 2,192	6.33%	U: 4,865	14.05%
Sept									4,219	12.19%
									D: 2,667	7.70%
									U: 1,552	4.48%

Supplementary Table S7 List of DEGs (FDR < 0.05, LFC cutoff = 0) identified from 2009, and 2015 and 2016 RNA-Seq data, respectively.

The table was uploaded as a separate Excel file.

Supplementary Table S8 GO terms enriched from the DEGs identified in three developmental transitions.

The table was uploaded as a separate Excel file.

Supplementary Table S9 Annotations of the 48 genes in the reproduction and pollination related GO categories.

***Populus* ID**	***Arabidopsis* ID**	**Annotation**
Potri.011G039400<br>Potri.011G039200<br>Potri.011G038800<br>Potri.004G027300<br>Potri.011G037900<br>Potri.004G027400<br>Potri.011G039100<br>Potri.011G039000<br>Potri.011G039300	AT1G11330	S-locus lectin protein kinase family protein
Potri.011G035200<br>Potri.004G028600<br>Potri.011G036600<br>Potri.011G034300<br>Potri.011G033800	AT1G11340	S-locus lectin protein kinase family protein
Potri.013G115800	AT1G34300	Lectin protein kinase family protein
Potri.013G095800<br>Potri.013G094000<br>Potri.013G096400<br>Potri.013G121000	AT2G19130	S-locus lectin protein kinase family protein
Potri.014G086900	AT4G00340	Receptor-like protein kinase 4
Potri.019G119900<br>Potri.019G120000	AT4G03230	S-locus lectin protein kinase family protein
Potri.011G128900<br>Potri.011G125000<br>Potri.001G414200	AT4G21380	Receptor kinase 3
Potri.011G037600<br>Potri.011G037300<br>Potri.005G014700<br>Potri.004G027800<br>Potri.005G014900<br>Potri.001G134900<br>Potri.011G037400	AT4G21390	S-locus lectin protein kinase family protein
Potri.T022900<br>Potri.001G411700<br>Potri.001G412100<br>Potri.001G409400<br>Potri.T023200<br>Potri.001G412000<br>Potri.T023000<br>Potri.T023900<br>Potri.T021800<br>Potri.001G412300<br>Potri.001G410800<br>Potri.011G129000<br>Potri.011G125200<br>Potri.011G128800	AT4G27290	S-locus lectin protein kinase family protein
Potri.008G016900	AT5G03700	D-mannose binding lectin protein with Apple-like carbohydrate-binding domain
Potri.002G086500	AT5G35370	S-locus lectin protein kinase family protein

Supplementary Table S10 Correlations among the five clusters identified by fuzzy c-means clustering.

**Cluster No.**	**1**	**2**	**3**	**4**	**5**
**1**	1.00	-0.87	0.83	-0.89	-0.70
**2**	-0.87	1.00	-0.51	0.61	0.26
**3**	0.83	-0.51	1.00	-0.99	-0.88
**4**	-0.89	0.61	-0.99	1.00	0.85
**5**	-0.70	0.26	-0.88	0.85	1.00

Supplementary Table S11 Number of genes identified in each of the four clusters using fuzzy c-means clustering. M indicates membership score, which was used for filtering genes for downstream analysis.

**Cluster No.**	**No. of Genes**						
	**Total**	**M ≥ 0.9**	**0.8≤M <0.9**	**0.7≤M <0.8**	**0.6≤M <0.7**	**0.5≤M <0.6**	**M <0.5**
**1**	3,548	56	572	667	593	584	1,076
**2**	1,770	0	0	0	144	328	1,298
**3**	2,737	0	87	330	502	625	1,193
**4**	3,084	0	94	380	518	690	1,402
**5**	3,276	0	289	541	620	605	1,221
**Total**	14,415	56	1,042	1,918	2,377	2,832	6,190

Supplementary Table S12 List of genes in each of the five clusters.

The table was uploaded as a separate Excel file.

Supplementary Table S13 Sequence motifs enriched from each cluster using MEME and HOMER, respectively.

No.	Motif Finder	Cluster 1	Cluster 3	Cluster 4	Cluster 5
1	MEME	MCCCCCTCCG	CCWCCGGG	STCGCGCG	GTCACCGGAC
2		GCCMGCGGC	CKAGMYTAG	CACGCTGGSCC	YGCGCCAA
3		CGGCTCCSTG	RASWTCGSG	CTCCGAMSCTCKTCG	TYGGACGRGT
4		GGACGCTCGCTGG	TCCTGTCG	AKGCCMGTS	AKGGCCCG
5		GGRRTKCCGCGCC	CCTGGTCG	WGGMCSGTC	AKGGCCCG
6		CCGGGCCY	ACCASRKRCMMRCGC	GCACKTGCSGG	AGGGCCCG
7		CRWCCGGC	YSSGCGCWGTTTMR	TAKGGMCC	CCRTGCGG
8		TYGAGCTGTY	CSGSGAYTG	CGSGMAAGWGSTC	CGWCACGG
9		KCRAKCWGTC	WKGAWWCG	MCTGCAACHARSCAG	CTCGGGGASC
10		GMCARGGCG	SSSRGTRYKM	CCSRCWAYCTAGCC	GGTGCGGG
11	HOMER	ATAATTACGT	TACGTACGAT	AACATTAACC	YRTGACCCGG
12		GATAATTACG	CCGTCTTTGA	CCAAGCGCCA	TAACCCGGGT
13		CGCAAGCTAG	CGTCTTTGAG	CGCCACGTGT	GTTRACCCGG
14		TGAACTAGTC	CCCGTGTTAA	GAAAGTGTAT	ACCCGGGTCA
15		ATTTAATAAC	CTTATTAWYT	AGTATATATA	SGTTRACCCG
16		ATTCCAGGCTGA	GACACGTCTTTG	TAGTATATATAC	GAAAAACTATTG
17		TAGTTATTAATA	GACAGGGTAAAA	TATTTAGCAATC	ATRACCCGGGTC
18		TATAGTTATTAA	AATATGTAGGAG	TTTCAATGTGGG	GACCCGGTCAAA
19		TAGTTTTTAAAC	AGTTAACTCATT	ATTTAGTAATCT	CCSGGTYGACCC
20		MWAGTTTTTAAA	TTTTGACCGGGT	ATTGAAAAGATA	TATAAAATTCAT
21		CSAKGCTGATRAST	AAATATGTAGGAGT	TTATCTGAAATATC	ATTTATGTACAAAA
22		GTTGATCGCTTCAA	TATTCTCGAGTCCC	ATATTCAGCAATCT	CTTTACCGACTATA
23		GMTTGACTTGGCWA	AGGASAKTACAAYT	AATATTTAGTAATC	ATGTATAGGTATTA
24		AAGTTAACAACCAT	GWCACACMTWTGTG	AAAGTATATATACT	TTATGTTTTTGTCT
25		ACCATRTAAGCTTY	SGGGTTTTGACCGG	YATCTAAAATATCT	AACCACACTTCCAA
26		TTCCAGGCTGATGACT	GACACGVCTTTGAGCV	ATATYTAGCAATCTAG	AGTTTGAAGAAGAAAC
27		TTGATCGCTTCAAAGC	GATTAAAAATGTGTAG	TGAAATATCTTATGCT	TYAATRTATAKGTRTT
28		TTTCTTTGATCTTTCA	GATTAAAAATATGTAG	AATATCTTATGCTCAT	TTTTTATCTTTATCTA
29		TGACTTGGCTAATTCA	WACTSRCACGTGTKAS	TAAATAAAACAAGGAA	ACAACCACACTTCCAA
30		GTGRRATTAGTYGAGG	TTTAAATATATATTTA	ATCTGAAATATCTTWT	AACTCRTGACCYRGGT
31		CGAGTTAA	TCATGTTA	GGCCCGTG	CGACCCGG
32		TTAACTTT	CATGTTAT	CGTGGGAT	GACCCGGT
33		GTTAATTT	WATAACAT	GTGGGATT	DGACCSGG
34		BAAGTTAA	TGGTTATT	ATTGGGAT	ATGACCCG

Supplementary Table S14 Comparison of high-confidence motifs against *Arabidopsis* motif database ArabidopsisDAP (v1).

The table was uploaded as a separate Excel file.

Supplementary Table S15 Gene models uniquely identified in age-dependent leaf senescence, seasonal leaf senescence, shared between senescence and bud dormancy, and between leaf senescence and cold acclimation, respectively.

The table was uploaded as a separate Excel file.

Supplementary Table S16 GO terms enriched from the DEGs shared between senescence and bud dormancy, and between leaf senescence and cold acclimation, respectively.

The table was uploaded as a separate Excel file.
